# Personal Continuity of Care in a University-Based Primary Care Practice: Impact on Blood Pressure Control

**DOI:** 10.1371/journal.pone.0134030

**Published:** 2015-07-27

**Authors:** Nik Sherina Hanafi, Adina Abdullah, Ping Yein Lee, Su May Liew, Yook Chin Chia, Ee Ming Khoo

**Affiliations:** 1 Department of Primary Care Medicine, University Malaya Primary Care Research Group (UMPCRG), Faculty of Medicine, University of Malaya, Kuala Lumpur, Malaysia; 2 Department of Family Medicine, Universiti Putra Malaysia, Serdang, Malaysia; University of Colorado Denver, UNITED STATES

## Abstract

Continuity of care is an important quality outcome of patient care. This study aimed to investigate the relationship between personal continuity and blood pressure (BP) control among the patients with hypertension in an academic primary care centre. Between January and May 2012, we conducted a retrospective review of medical records of patients with hypertension who had been followed up for at least 1 year in the Primary Care Clinic, University of Malaya Medical Centre, Malaysia. In this setting, doctors who provided care for hypertension included postgraduate family medicine trainees, non-trainee doctors and academic staff. Systematic random sampling (1:4) was used for patient selection. BP control was defined as less than 130/80 mm Hg for patients with diabetes mellitus, proteinuria and chronic kidney disease and less than 140/90 mm Hg for all other patients. Continuity of care was assessed using the usual provider continuity index (UPCI), which is the ratio of patient visits to the usual provider to the total number of visits to all providers in 1 year. A UPC index of zero denotes no continuity while an index of one reflects perfect continuity with only the usual provider. We reviewed a total of 1060 medical records. The patients’ mean age was 62.0 years (SD 10.4). The majority was women (59.2%) and married (85.7%). The mean number of visits in a year was 3.85 (SD 1.36). A total of 72 doctors had provided consultations (55 postgraduate family medicine trainees, 8 non-trainee doctors and 9 academic staff). The mean UPCI was 0.43 (SD 0.34). Target BP was achieved in 42% of the patients. There was no significant relationship between BP control and personal continuity after adjustment for total number of visits. Continuity of care was not associated with BP control in our centre. Further studies are needed to explore the reasons for this.

## Introduction

Hypertension is a major health issue responsible for 4.5% of current global disease burden [1]. A Malaysian nation-wide survey showed that 43% of adults aged more than of 30 years have hypertension, and among those who were on treatment, only a quarter (26.3%) had controlled blood pressures (BPs) [2].

Factors contributing towards poor BP control can be categorised into system, doctor and patient factors [3]. One aspect that spans across these three factors is continuity of care. Continuity of care is widely regarded as an important component of quality health care and a core tenet of primary care [4]. Varying concepts have been developed to define and measure continuity of care [5,6]. In general, continuity of care can be defined as personal or care continuity. Personal continuity stresses on patients seeing the same healthcare provider at each visit, while care continuity stresses on continuity derived from the perspectives of organisation, guidelines and medical records. The latter does not necessitate seeing the same provider at each visit [7]. The concepts for continuity of care cover informational, chronological, geographical, interdisciplinary, and interpersonal continuity [8,9]. Continuity of care has a hierarchical dimension with informational continuity being ranked the lowest, followed by longitudinal continuity and interpersonal continuity as the highest level of continuity [10].

There are conflicting evidence on the effect of continuity of care on health indices and outcome. Continuity of care has been reported to result in improved doctor–patient relationship [4], long-term appointment keeping [11], patient satisfaction [12], physician satisfaction [13] and drug compliance [14]. It also has been shown to result in reduced hospitalisation risks, emergency department visits and health care costs for several chronic diseases [15] as well as better awareness of hypertension and adherence to medication [16]. However, there are also the studies that showed continuity of care did not affect assessment of chronic disease and outcomes [17,18].

Continuity of care can be influenced by the type of practice. Primary care in Malaysia is provided by a dual system of public and private clinics. Public clinics provide subsidised health services for civil employees and pensioners as well as for schoolchildren. Private clinics, on the other hand, operate on a fee-for-service basis and are usually solo or small group practices where personal continuity is better maintained [19]. Continuity of care is also affected in academic primary care centres, where staff rotation and teaching duties influence personal continuity [20,21]. This study was carried out to investigate the relationship between personal continuity of care and BP control in a public university-based primary care centre.

## Materials and Methods

### Study design

This was a retrospective longitudinal cohort study conducted by reviewing 1060 medical records of adult patients with hypertension.

### Setting

This study was conducted in the Primary Care Clinic, University of Malaya Medical Centre in urban Kuala Lumpur, Malaysia. This clinic is a university-based primary care centre with approximately 70 doctors. Patients attend scheduled clinic appointments for chronic disease management under individual personal doctors. If the personal doctor was absent, the patient would be seen by another doctor. Informational continuity is maintained though the means of paper-based medical records. There were three categories of doctors: family medicine trainees undergoing a 4-year postgraduate family medicine residency programme, non-trainee doctors who provide full-time clinical service and academic staff. The academic staffs were primary care physicians with postgraduate qualifications who provide clinical service, teach undergraduate and postgraduate students and undertake clinical research.

### Sampling

All medical records of patients with hypertension who attended the clinic between January and May 2012 and who had been on follow-up at the clinic for at least 1 year were used as the sampling frame. Prior to the appointments, research assistants manually went through each paper-based record to identify patients with hypertension. Patients fitted the inclusion criteria if the diagnosis of hypertension was documented in the clinical notes, have had hypertension for at least 1 year or who were on treatment for hypertension. These records were tagged for identification. Approximately 4000 patients with hypertension attended their follow-up appointments during this period. At the end of the clinic session, all tagged records were recollected. Records were randomly selected based on a 1:4 systematic selection. A total of 1060 records were included.

### Operational definition of variables

BP value: BP value was the average of mean systolic blood pressure (SBP) and diastolic blood pressure (DBP) values recorded in the medical records in all visits over the past 1 year. All BP readings were individualised.

Level of control: BP control was defined as achieving a BP of less than 130/80 mm Hg in patients with diabetes mellitus, proteinuria and chronic kidney disease, and less than 140/90 mm Hg for all other patients based on the Malaysian clinical practice guideline on the management of hypertension [22].

Diabetes mellitus: Based on the documented diagnosis in patients’ medical records and/or fasting blood sugar (FBS) ≥ 7.0 mmol/L.

Proteinuria: Based on the laboratory results documented in patients’ clinical notes.

Chronic kidney disease: Based on the documented diagnosis in patients’ clinical notes.

### Data collection

An electronic self-designed data collection form was used to collect the following information from the paper-based medical records: socio-demographic data, clinical assessment profile, outcome indicators (the proportion of patients achieving target BP control) and names of attending as well as personal doctors. Accuracy of data was ensured by double data entry and random data audit by researchers. Data collection did not involve patient contact.

### Measurement of continuity of care

There are various methods of measuring continuity of care. These include the usual provider continuity index (UPCI), continuity index, continuity of care (COC) index, Gini index, likelihood of continuity and sequential continuity [23,24]. We operationalised continuity of care as patients seeing the same personal doctor at each visit, measured using the usual provider continuity index (UPCI) [23,25].

$$\text{UPCI}=\frac{\text{Number of patient visits to the personal doctor}}{\text{Total number of visits}}$$

The UPC index was chosen as it has been designed to quantify visit patterns when patients were assigned to one personal doctor. The index ranges from 0 to 1 with 1 denoting perfect personal continuity.

### Data analysis

Data were analysed using the statistical package for social sciences (SPSS) version 18 (Chicago, Illinois, USA). Continuous data were expressed as mean ± standard deviation (SD). Categorical variables were expressed as absolute (number) and relative frequencies (percentages). Independent *t*-test was used to compare continuous variables while Pearsons *χ* ^2^ test was used for the comparison of categorical variables. Missing values were excluded from analysis. A linear regression model was performed to determine the association between factors assessed in the clinical assessment and UPCI. A *p*-value of <0.05 was considered statistically significant.

### Ethics statement

Ethics approval was obtained from the University of Malaya Medical Ethics Committee (Reference number: 890.14). Data were anonymised to ensure patient confidentiality.

## Results

We reviewed a total of 1060 medical records. The patients’ mean age was 62.0 years (SD 10.4). The majority was women (59.7%) and married (85.7%) (Table 1). The mean duration of hypertension was 8.5 years (SD 6.4).

**Table 1:** Patients sociodemographic profile.

Profile	*n* (%)
*Gender (n = 1060)*	
Male	427 (40.3%)
Female	633 (59.7%)
*Ethnicity (n = 1060)*	
Malay	323 (30.5%)
Chinese	509 (48.0%)
Indian	218 (20.6%)
Others	10 (0.9%)
*Marital status (n = 1030)*	
Single	64 (6.2%)
Married	894 (86.8%)
Widow/widower	72 (7.0%)

A total of 72 doctors provided the consultations (55 postgraduate family medicine trainees, eight non-trainee doctors and nine academic staff). The majority of patients were seen by postgraduate trainees (*n* = 795, 75%). The number of visits ranged from one to 13 with a mean of 3.85 (SD 1.36) visits. The majority of patients had a total of three visits in past 1 year (*n* = 352, 33.2%).

The mean UPCI was 0.43 (SD 0.34) with a range of 0.00 to 1.00. A total of 144 patients (13.6%) had perfect personal continuity (UPCI of 1.00), with total visits ranging from 1 to 6 (Table 2).

**Table 2:** Frequency of visits among patients with provider continuity of 1.00 over 1 year.

Number of visits in the past 1 year	Frequency *n* (%)
1	1 (0.7)
2	44 (30.6)
3	61 (42.4)
4	31 (21.5)
5	6 (4.2)
6	1 (0.7)

The all-group mean blood pressures were SBP 136.2 (SD 11.2) mm Hg and DBP 79.5 (SD 6.3) mm Hg. Target BP was achieved in 42.0% (*n* = 445) of patients. Among the poorly controlled group, the mean SBP and DBP were 142.2 (SD 9.6) and 81.6 (SD 6.5) mm Hg respectively; while the mean SBP and DBP for the controlled group were 127.7 (SD 6.4) and 76.6 (SD 4.8) mm Hg respectively. The differences for both SBP and DBP were statistically significant [SBP mean difference 14.7 (95% CI 13.7–15.7) and DBP mean difference 5.0 (95% CI 4.3–5.7)].

There was no statistically significant difference in BP control with the average number of visits per year. There were no significant associations between mean SBP and mean DBP with UPCI (r = −0.005, *p* = 0.866 and r = −0.012, *p* = 0.697 respectively) (Table 3).

**Table 3:** Number of visits and personal continuity among patients with controlled and uncontrolled blood pressure.

	BP controlled (n = 445, 42.0%)	BP not controlled (n = 615, 58.0%)	*p*-value
Number of visits per year (mean)	3.70	3.95	0.302
UPCI (mean)	0.42	0.43	0.233

Multivariate analysis showed no significant association between BP control and personal continuity after adjusting for total number of visit (Table 3). Total number of visits was negatively associated with BP control indicating that the higher the number of visits, the worse the BP control (Table 4).

**Table 4:** Relationship between blood pressure control and UPCI.

Model	Standardised Coefficients	*t*	sig	95% I	
Total number of visits	−0.094	−3.043	0.002	−0.056	−0.012
UPCI	−0.020	−0.655	0.512	−0.117	−0.058

## Discussion

The key finding of this study, where the mean UPCI was 0.43, was the absence of relationship between personal continuity and BP control. This finding appeared to be inconsistent with our original hypothesis that personal continuity of care is associated with better BP control. The lack of association remained after adjusting for total number of visits. Possible reasons to account for this include: 1) the continuity of care was provided by information continuity in this centre and 2) clinical management did not differ between physicians because of training and standardisation of practice. Similarly, data from the NHANES III found no relationship between continuity of care and recognition of hypertension, since BP is typically measured regardless of the degree of continuity provided [26].

An earlier study had shown that the continuity of care in hypertension was positively associated with better BP control [27]. However, more recent studies done in academic settings similar to ours did not show any association between BP control and continuity of care [20,28]. This could be explained by several reasons. First, the mean SBP of our study population was 136 mmHg, which was already below the target SBP. Hence, a further reduction in BP might be difficult to attain even with greater personal continuity. Secondly, the emphasis on clinical practice guidelines in our centre meant that doctors provided standardised care, which minimised the effects of personal continuity.

Several other studies have shown that continuity of care is positively associated with better outcomes. However, these studies looked at delivery of preventive services and reduction of hospitalisation which were more easily achievable in a shorter duration of time [24].

This study indicated that in a setting where providers function as a team and information continuity is maintained through the means of medical records, personal continuity doesn’t result in a significant difference to BP control. Further studies are required to explore the effects of other factors such as information continuity and standards of care.

### Strength and limitations

The strength of our study was that it had a larger sample size of patients and providers compared to the previous studies [20,28]. This provided a more reliable result on the relationship of personal continuity and BP control. The sampling of our study was done systematically from a larger population of more than 4000 patients who attended the clinic during the study period. The limitation was that this study was a retrospective record review which depended on the documentation by the clinical care providers.

## Conclusion

Personal continuity was not associated with BP control. This is likely to be due to the contribution of information continuity and standardised care in a setting where information continuity is maintained.
